# Maslinic Acid Protects against Streptozotocin-Induced Diabetic Retinopathy by Activating Nrf2 and Suppressing NF-*κ*B

**DOI:** 10.1155/2022/3044202

**Published:** 2022-02-28

**Authors:** Nasser A. Alsabaani, Osama M. Osman, Mohamed A. Dallak, Mohamed D. Morsy, Hassan A. Al-Dhibi

**Affiliations:** ^1^Department of Ophthalmology, College of Medicine, King Khalid University, Abha, Saudi Arabia; ^2^Department of Physiology, College of Medicine, King Khalid University, Abha, Saudi Arabia; ^3^Department of Physiology, College of Medicine, Menoufia University, Shebeen El-Kom, Egypt; ^4^Division of Vitreoretinal Surgery and Uveitis, King Khalid Eye Specialist Hospital, Riyadh, Saudi Arabia

## Abstract

This study tested the protective effect of maslinic acid (MA) against diabetic retinopathy (DR) in rats with type 1 diabetes mellitus (T1DM) and investigated possible mechanisms of action. DM was introduced by streptozotocin (STZ) (65 mg/kg, i.p.). Control and STZ (T1DM) were divided into 2 subgroups, which received either the vehicle or MA (80 mg/kg). Serum, pancreases, and retinas were collected for further use. MA significantly reduced fasting glucose levels in the control and T1DM rats but enhanced fasting insulin levels and partially increased the size of the islets of Langerhans and the number of *β*-cells in T1DM rats. In addition, MA significantly improved the retina structure by preventing the reduction in the area between the inner and outer limiting membranes (ILM and OLM, respectively) and increasing the number of cells forming the ganglion cell layer (GCL), inner nuclear layer (INL), and outer nuclear layer (ONL). Associated with these effects, MA significantly reduced the total levels of tumor necrosis factor-*α* (TNF-*α*) and interleukin-6 (IL-6), as well as the nuclear levels of NF-*κ*B p65, mRNA levels of Bax, and protein levels of cleaved caspase-3 in the retinas of T1DM rats. However, MA significantly lowered levels of reactive oxygen species (ROS) and malondialdehyde (MDA) but significantly increased the nuclear levels of Nrf2, protein levels of Bcl2, and total levels of superoxide dismutase (SOD) and reduced glutathione (GSH) in the retinas of the control and T1DM rats. In conclusion, MA prevents DR by antioxidant potential mediated by the activation of Nrf2.

## 1. Introduction

Diabetes mellitus (DM) is a chronic world pandemic disorder and is the 4th leading cause of death worldwide due to the development of several microvascular and microvascular complications [1]. According to the most recent studies, the global prevalence of DM was 9.3% in 2019, a percent that is projected to increase to 10.2% and 10.9% by the years, 2030 and 2045, respectively [2]. According to the etiology, DM can be either type 1 DM (T1DM) that is characterized by complete loss of insulin or T2DM that is associated with insulin resistance (IR) and peripheral insulin ineffectiveness [1].

Diabetic retinopathy (DR) is the most common microvascular complication and common eye disease in the majority of patients with T1DM and is seen in more than 60% of patients with T2DM [3]. At the clinical level, DR is a major cause of visual impairment and blindness among diabetic patients [4, 5]. The disease can be classified into two major types according to the time of the development, an early nonproliferative DR (NPDR), and a more advanced later type, the proliferative one (PDR). Damaged blood-retina barrier (BRB) that is associated with increased vascular permeability and leakage, micro-aneurysms, retinal haemorrhage, macular edema, and exudates are the initial events of DR and usually occur early during the NPDR stage [6, 7]. On the other hand, severe retinal ischemia, abnormal proliferation of the endothelial and epiretinal fibrovascular cells, increased thickness of the basement membrane, capillary closure, neovascularization, contraction of fibrovascular epiretinal membrane (ERM), and detachment of the retina represent the major events leading to blindness, which are more common during the PDR stage [8, 9].

However, the DR is a vascular oxidant and inflammatory disorder in which locally produced reactive oxygen species (ROS) and inflammatory cytokines and chemokines mediate their pathogenesis and are the major triggers for all other well-known damaging events [10–14]. However, both hyperglycaemia and systemic inflammation play significant roles in this process but seem to be dependent on the type of diabetes. Indeed, both hyperglycaemia and systemic inflammation are major triggers of DR in T2DM patients, whereas systemic inflammation has a limited role in T2DM-induced DR [15]. Within this view, increased influx of the systemic inflammatory cytokines is an independent factor that can initiate the damage in the diabetic retina by damaging the BRB, increasing the vascular permeability, exaggerating ROS generation, activating microglial cells and macrophage infiltration, and enhancing pericytes and neural apoptosis [10, 16, 17].

On the other hand, hyperglycaemia alone is an independent factor that triggers all the damaging pathways in the retina by increasing the production of ROS and subsequent activation of inflammation and apoptosis [10, 13, 18]. Within this view, sustained hyperglycaemia can stimulate the retinal generation of ROS by increasing mitochondria oxidative phosphorylation, activation of the cytosolic NADPH oxidase (NOX), depleting antioxidants, and autoxidation [10, 13, 19]. These ROS can further amplify the production of other ROS and activate several inflammatory and apoptotic pathways through stimulating several key mechanisms such as hexose monophosphate, polyol, protein kinase C (PKC), and advanced glycation end products (AGE) pathway [13]. Interestingly, the use of pharmacological antioxidant therapy or transgenic approaches to stimulate the retina antioxidant systems has indicated that hyperglycaemia-induced ROS is the major mechanism responsible for the activation of all damaging inflammatory and apoptotic responses in the diabetic eye [10, 19–24]. Therefore, drugs with antioxidant potential may provide a novel therapy to prevent or treat DR in patients.

On the other hand, much attention is currently given to triterpenoids in treating chronic and metabolic disorders. Maslinic acid (MA) is a pentacyclic triterpene that is isolated from several fruits and vegetables (e.g., olive skin, basil, and mustard). MA is pharmacologically safe and has been potent antioxidant and inflammatory properties [25]. Experiments from several animal models, as well as from in vitro studies, have confirmed these protective effects and showed that MA can cardiac, neural, renal, bone, and hepatic damage by attenuating oxidative stress and inflammation [25–33]. In this regard, the antioxidant potential of MA has been attributed to its ability to scavenge ROS, upregulate enzymatic and nonenzymatic antioxidants, suppress ROS-generating enzymes (e.g., NADPH oxidase (NOX) and iNOS), and stimulate the nuclear accumulation and DNA binding activity of the nuclear factor erythrocyte 2-related factor 2 (Nrf2) and subsequently upregulation of the heme-oxygenase-1 (HO-1) [10, 25–29]. On the other hand, several mechanisms underlie the anti-inflammatory effect of MA including suppression of the activation of nuclear factor kappa-light-chain-enhancer of activated B cells (NF-*κ*B), downregulation of the transcription of inflammatory cytokines and adhesive molecules, suppression of cyclooxygenase-2 (COX-2), and the generation of inflammatory prostaglandins [28, 30–33].

Of note, MA showed a potent hypoglycaemic effect and was able to prevent renal, cardiac, and hepatic damage in streptozotocin (STZ)-induced T1DM in rats, by suppressing ROS and concomitant upregulation of the antioxidant enzymes, superoxide dismutase (SOD), and glutathione peroxidase (GPX) [34]. Yet, the protective effect of MA against diabetic retinopathy was not tested.

Therefore, in the study, we have evaluated the ability of MA to prevent diabetic retinopathy in STZ-induced T1DM in rats and investigated some of its mechanisms of action.

## 2. Materials and Methods

### 2.1. Animals

Twenty-four adult male Wistar rats weighing 230 ± 10 g were obtained from the animal house at the College of Medicine of King Khalid University (KKU), Abha, Kingdom of Saudi Arabia. All rats were housed in plastic cages (4 rats/cage) under fixed conditions (12-h light/dark cycle, 23 ± 1°C, and 60% humidity) and had free access to their diets and drinking water. All procedures and protocols followed in this study were approved by the ethical committee of the College of Medicine at KKU, which follows the guidelines of Animals in Research: Reporting *In Vivo* Experiments (ARRIVE) [35] and those issued by the National Institutes of Health for the care and use of laboratory animals (NIH Publications No. 8023, revised on 1978).

### 2.2. Induction of T1DM

STZ is the best known chemical drug that causes T1DM by causing oxidative damage of the pancreatic *β*-cells [36]. In rats, the administration of a single dose of STZ (60–65 mg/kg) causes T1DM and results in DR with enhanced retinal oxidative stress and inflammation in approximately 4–6 months postinjection [37, 38]. Therefore, rats were administered a single i.p. dose of STZ (Cat 142155, Sigma-Aldrich, MO, USA) (65 mg/kg dissolved in 0.5 M citrate buffer), and blood glucose levels were monitored for the next 3 days. At the end of day 3, blood samples were withdrawn from the tail and used to check glucose levels (twice/day). Rats showing fasting glucose levels higher than 300 mg/dl were considered to have T1DM and were included in the experimental design as shown below.

### 2.3. Experimental Design

One day after confirming T1DM in rats, control nondiabetic and diabetic rats were divided into 4 groups (each of 8 rats) as follows: (1) control rats—aged-matched nondiabetic rats and received 5% carboxymethyl cellulose (CMC) as a vehicle; (2) control + MA—nondiabetic rats and received MA dissolved in 5% CMC at a final concentration of 80 mg/kg; (3) T1DM—were diabetic rats and received 5% CMC; and (4) T1DM + MA—were diabetic rats and received MA solution at a final dose of 80 mg/kg. Treatment with the MA at the selected dose (80 mg/kg) or vehicle was given once every three days as shown by Mkhwanazi et al. [34], an effective dose to prevent STZ-induced oxidative stress, inflammation, and damage in rat's heart, kidney, and liver. All treatments were conducted for 6 months. Food and water intake, as well as body weights, were measured weekly.

### 2.4. Serum and Tissue Collection

By the last day of the experiments, rats of all groups were administered 1 ml/kg ketamine/xylazine mixture containing 80 mg ketamine hydrochloride (Cat K-101, Sigma-Aldrich, MO, USA) and 12 mg xylazine hydrochloride (Cat X-101, Sigma-Aldrich, MO, USA). Once the unresponsive to painful stimuli was confirmed (45–60 min), blood samples (1.5 ml) were collected from the heart into plain tubes. These tubes were centrifuged at 1300x g for 5 min to collect serum which further was stored at −20°C until use. Then, all rats were euthanatized by dislocation of the neck, and their eyes were dissected out, and freed from excess tissue and muscle in ice-cold phosphate-buffered saline (PBS). The retina of each eye was identified and isolated. Parts of the retina were fixed in 10% buffer formalin and used for the histological evaluation as indicated below. Other parts were frozen at −80°C for further use.

### 2.5. Measurement in the Serum

Serum glucose levels were measured using a rat's specific assay kit (Cat 81693; Crystal Chem, IL, USA). Serum insulin levels were measured using a rat's specific ELISA kit (Cat MBS2700141, MyBioSource, CA, USA). Serum levels of triglycerides were measured using an assay kit (Cat 10010303, Cayman Chemical, MI, USA). Serum levels of total cholesterol were measured using an assay kit (Cat STA-384, Cell Biolabs, CA, USA). Serum levels of low-density lipoprotein cholesterol (LDL-c) were measured using an assay kit (Cat 80069, Crystal Chemicals, USA).

### 2.6. Preparation of Tissue Homogenates and Biochemical Analysis

Parts of the retina were homogenized in 9 volumes of ice-cold PBS (pH = 7.4), centrifuged at 11000x g for 10 min. The supernatants were isolated, frozen at −80°C, and used later for the following analysis. Total levels of ROS and reactive nitrogen species (RNS) were measured using OxiSelect™ In Vitro ROS/RNS Assay Kit (cat STA-347, Cell Biolabs, CA, USA). The retinal levels of superoxide dismutase (SOD) were measured using a special ELISA kit (Cat MBS2707324, MyBioSource, CA, USA). Total levels of the reduced glutathione (GSH) were measured using a rat ELISA-based kit (Cat MBS046356, MyBioSource, CA, USA). Levels of tumor necrosis-*α* (TNF-*α*) (Cat ab46070, Abcam, UK). Levels of interleukin-6 (IL-6) were measured using an ELISA kit (Cat ab100713, Abcam, UK). Levels of the intracellular cell-adhesive molecule (ICAM) were measured using a rat's ELISA kit (Cat MBS267983, MyBioSource, CA, USA).

### 2.7. Isolation of the Nuclear Fraction and Measurement

The nuclear and cytoplasmic fractions of the frozen retina were prepared to form *n* = 6 retinas/group using a commercially available kit as per the manufacturer's instructions (Cat 78833; ThermoFisher, USA). Nuclear levels (activation) of NF-*κ*B p65 were measured by the TransAm assay (Cat 40596, Active Motif, Tokyo, Japan). The nuclear levels (activation) of Nrf2 were measured using the TransAM assay (Cat 50296 Active Motif, Tokyo, Japan). All procedures were performed in duplicate and following the manufacturers' instructions.

### 2.8. Real-Time Polymerase Chain Reaction (qPCR)

mRNA levels of some apoptotic markers were measured using the qPCR method. Primer sequences used to amplify Bcl2, Bax, and *β*-actin were designed and purchased for ThermoFisher. The primer pairs for Bax (Acc. No. NM_017059) were F: ATGGAGCTGCAGAGGATGATT and R: TGAAGTTGCCATCAGCAAACA (97 bp); for Bcl2 (U34964.1) were F: TGGGATGCCTTTGTGGAACT and R: TCTTCAGAGACTGCCAGGAGAAA (73 kDa); and for *β*-actin (NM_031144.2) were F: TACCCAGGCATTGCTGACAG and R: AGCCACCAATCCACACAGAG. RNAs were isolated from all retina tissues using the HeneJet isolation kit (Cat K0731, ThermoFisher). The first-strand cDNA was synthesized using the Verso synthesis kit (Cat AB1453 A, ThermoFisher). RNA isolation and cDNA synthesis were performed as per each kit's instructions. The mRNA amplification reaction was performed in the BioRad CFX96 system (USA) using Ssofast Evergreen supermix master mix and as per the manufacturer's instructions. The following ingredients were added to the reaction mixture (10 *µ*l): template cDNA (1 *μ*l/500 ng), a forward primer of each target (0.2 *μ*l/200 nm/reaction), reverse primer of the target (0.2 *μ*l/200 nm/reaction), master mix (5 *μ*l), and nuclease-free water (3.6 *μ*l). The amplification was performed using the following steps: (1) 1 cycle heating for 30-sec°C, (2) 34 cycles of denaturation for 30 sec at 95°C, (3) 34 cycles of annealing for 30 sec at 60°C for 30 sec, and (4) a final melting Step (1 cycle) for 1 sec and 95°C. The relative expression of both Bcl2 and Bax were normalized to the mRNA levels of the reference gene, *β*-actin using the software using the 2−^ΔΔCt^ method.

### 2.9. Western Blotting

Total protein in the cytoplasmic and the nuclear fraction determined using the quantity-pro ABC assay kit (Cat QPBCA-1 KT, Sigma-Aldrich, MO, USA). Samples were diluted in the loading dye to prepare final protein concentrations of 2 *µ*g/*µ*L. All samples were boiled for 5 min and then loaded in the separation wells at a final concentration of 40 *µ*g/ml. Samples were separated using the SDS-PAGE for 2 h at 100 volts for 1 h and then transferred to nitrocellulose membranes for another 2 h at 80 v. Membranes were then blocked using 5% skimmed milk diluted in the washing buffer (1X TBST) for 30 sec at room temperature with shaking. The membranes were then washed with the TBST buffer 3 times each of 10 min at room temperature and then incubated with the primary monoclonal antibodies against Bcl2 (Cat sc-7382, 1 : 1000, 26 kDa), Bax (Cat sc-7480, 1 : 1000, 23 kDa, lamin A (sc-518013, 1 : 1000; 69 kDa) (Santa Cruz Biotechnology, USA), cleaved caspase-3 (Cat 9661, 1 : 500, 17/19 kDa), Nrf2 (Cat 12721, 1 : 500, 100 kDa), and *β*-actin (Cat. No. 47778, 1 : 10000, 45 kDa) (Cell Signaling Technology, USA). The membranes were then washed with the TBST buffer 3 times (each of 10 min) and then incubated with shaking with the corresponding secondary antibodies for 2 h at room temperature. After washing, the interaction between the 1st and 2nd antibodies was developed by incubating with 2 ml of reagents A and B of the enhanced chemiluminescence ECL pierce kit (Cat 32109, Thermo Fisher) for 3 min. After development, all gels were scanned using the C-Di Git blot scanner (LI-COR, USA), and all band intensities were analyzed using the provided software. Expression of Bax, cytochrome-c, and cleaved caspase-3 was normalized to the corresponding expression *β*-actin, whereas the expression of Nrf2 and NF-*κ*B p65 were normalized to the corresponding expression of lamin A.

### 2.10. Histological Studies

Freshly collected retina parts were fixed in 10% buffered formalin for 24 h. All tissues were deparaffinized and rehydrated using xylene and descending alcohol concentrations (100%, 90%, and 70%). The tissues were cut at 3–5 *µ*M and then stained with the Harris hematoxylin containing glacial acetic acid, rinsed with deionized water, destained with HCL/70% ethanol (1: 400 v/v), and then stained with Eosin Phloxine stain. All the slides were dehydrated with 95% and 100% ethanol and with xylene. A drop of mounting media was added to every slide, covered with a coverslip, and left overnight to dry. All images were examined and captured under a light microscope at 200X.

### 2.11. Statistical Analysis

All analysis and graphing were performed on the GraphPad prism analysis software (version 8). Normality was tested using the Kolmogorov-Smirnov test. The comparison between all data was made using the 1-way ANOVA followed by Tukey's *t*-test as a post hoc test. A *P* ˂ 0.05 was considered the levels of significance. All data were presented as means ± the standard deviation (mean ± SD).

## 3. Results

### 3.1. MA Reduces the Weight Gain and Improves the Hyperglycaemia in T1DM-Induced Rats

Final body weights, average weekly food and water intake, and fasting insulin levels were not significantly varied between control and MA-treated rats (Table 1). However, fasting glucose levels were significantly decreased in MA-treated rats as compared to control rats (Table 1). On the other hand, final body weights, food intake, water consumption, and fasting glucose levels were significantly decreased, but fasting insulin levels were significantly reduced in T1DM-induced rats as compared to control rats, but all were significantly reversed in T1DM + MA-treated rats (Table 1).

### 3.2. MA Preserves the Pancreatic Tissues in T1DM-Induced Rats

**Figure 1:**
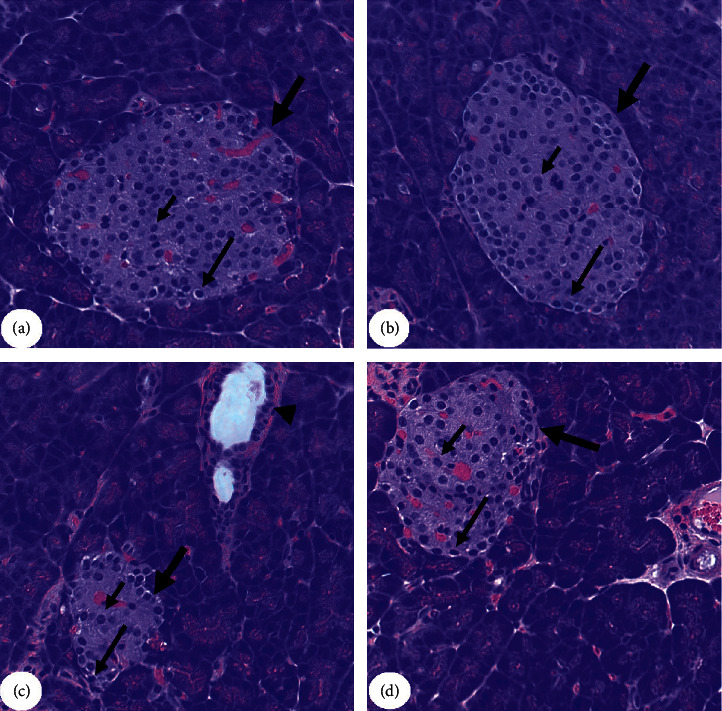
Photomicrographs of the pancreases from all groups of rats. A & B represent control and MA-treated rats. In both images, the islets of Langerhans (thick arrow) appeared circular and were of large size. The exocrine (*α*)-cells were located at the periphery and had normally small cells with dark nuclei (long thin arrow), whereas the endocrine *β*-cells (short arrow) are located centrally with larger with light nuclei. C represents a T1DM rat in which the islets of Langerhans appeared smaller (thick arrow) with a severe reduction in the number of the *β*-cells (short arrow). Not the dilated blood vessel with increased inflammatory cells (arrowhead). D represents T1DM + MA-treated rats and showed an increase in the size of the islet of the Langerhans and the number of *β*-cells (short arrow) (200X).

Control and MA-treated rats showed normal and similar pancreases structures that are characterized by intact peripheral darkly stained, small exocrine (*α*)-cells and central, large, lightly stained *β*-cells (Figure 1). The size of the islets of the Langerhans, as well as the number of *α* and *β*-cells, was significantly reduced in the T1DM-treated rats as compared to control rats (Figure 1). However, although remained slightly smaller than those of the control and MA-treated rats, an obvious enlargement in the size of the islets of the Langerhans with a clear increase in the number of both *α-* and *β*-cells was observed in the pancreatic tissues of the T2DM + MA-treated rats as compared to T1DM-treated rats (Figure 1).

### 3.3. MA Preserves the Retina Structure in T1DM-Induced Rats

**Figure 2:**
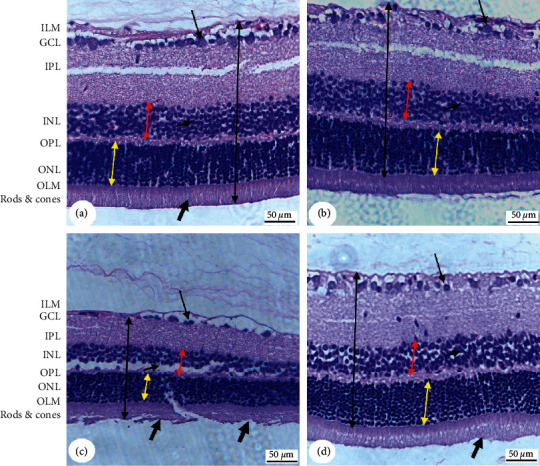
Histopathological photomicrographs of the retina of all groups of rats as stained with hematoxylin and eosin (H & E) staining. 200X. All layers of the retina including the inner limiting membrane (ILM), ganglion cell layer (GCL), inner plexiform layer (IPL), inner nuclear layer (INL), outer plexiform layer (OPL), outer nuclear layer (ONL), outer limiting membrane (OLM), and photoreceptors (rods and cones) were identified in all sections. A and B were taken from control and MA-treated rats and showed a normal thickness of all layers with an abundant cell number in the GCL (long arrow) and INL (short arrow) and intact photoreceptor layer (thick arrow) and ONL. Not the thickness between the ILM and OLM layers (black double arrow), ONL (yellow double arrow), and INL (red double arrow). (C) was taken from T1DM-induced rats and showed a significant reduction in the thickness. Besides, severe damage in the photoreceptor layer (Thick arrow) with severe loss of the majority of the cells in the GCL (long arrow) and INL (short arrow) was seen. (D) was taken from a T1DM + MA-treated rat and showed an almost normal retina structure with a preserved thickness of all layers and rods that were associated with an increased number of cells composing the INL (200X).

Normal retina tissues with intact inner limiting membrane (ILM), outer limiting membrane (OLM), and photoreceptors (rods and cones), as well as ganglion cell layer (GCL), inner plexiform layer (IPL), inner nuclear layer (INL), outer plexiform layer (OPL), and outer nuclear layer (ONL), were observed in the retina sections of both the control and MA-treated rats (Figures 2(a) and 2(b)). However, retinas from the T1DM-induced rats showed a significant reduction in the thickness of both the INL and ONL and in the distance between them (Figure 2(c) and Table 2). They also showed a significant reduction in the number of cells forming the GCL, INL, and ONL (Figure 2(c) and Table 2). As compared to T1DM rats, T1DM + MA-treated rats showed a significant improvement in the structure of the retina that is characterized by a significant increase in the thickness of the ILM and OLM with a concomitant improvement in the distance between them and in the number of cells composing them (Figure 2(d) and Table 2). In addition, T1DM + MA-treated rats had higher ganglionic cell numbers in their GCL as compared to T1DM-treated rats (Figure 2(d) and Table 2).

### 3.4. MA Suppresses Inflammation in the Retinas of the T1DM-Treated Rats

**Figure 3:**
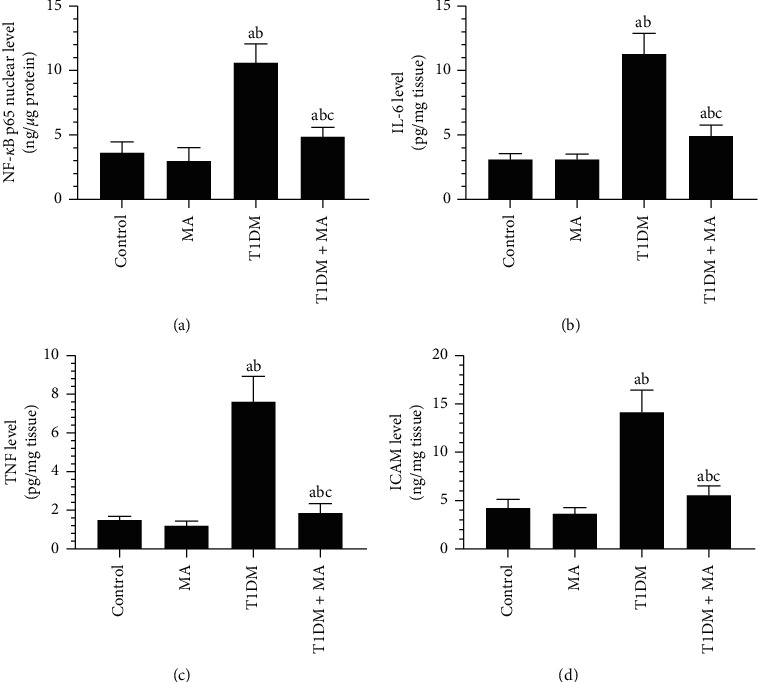
Levels of inflammatory markers in the retina of all groups of rats. Data were analyzed by 1-way ANOVA, and all results were presented as mean ± SD (*n* = 8). Values are considered significantly different at *P* < 0.05. ^a^: significantly different as compared to control rats. ^b^: significantly different as compared to MA-treated rats. ^c^: significantly different as compared to T1DM-induced rats.

No significant alterations in the nuclear levels of NF-*κ*B p65 nor the total homogenate levels of TNF-*α*, IL-6, and ICAM-1 were depicted between the control and MA-treated rats (Figures 3(a)–3(d)). The levels of all these inflammatory markers were significantly increased in the retinas of the T1DM-induced rats as compared to control rats (Figures 3(a)–3(d)). On the other hand, the nuclear activity of NF-*κ*B p65, as well as the total homogenate levels of TNF-*α*, IL-6, and ICAM-1, was significantly decreased in the retinas of T1DM + MA-treated rats as compared to T1DM-induced rats (Figures 3(a)–3(d)).

### 3.5. MA Suppresses Oxidative Stress and Stimulates Nrf2 in the Retinas of Both the Control and T1DM-Treated Rats

**Figure 4:**
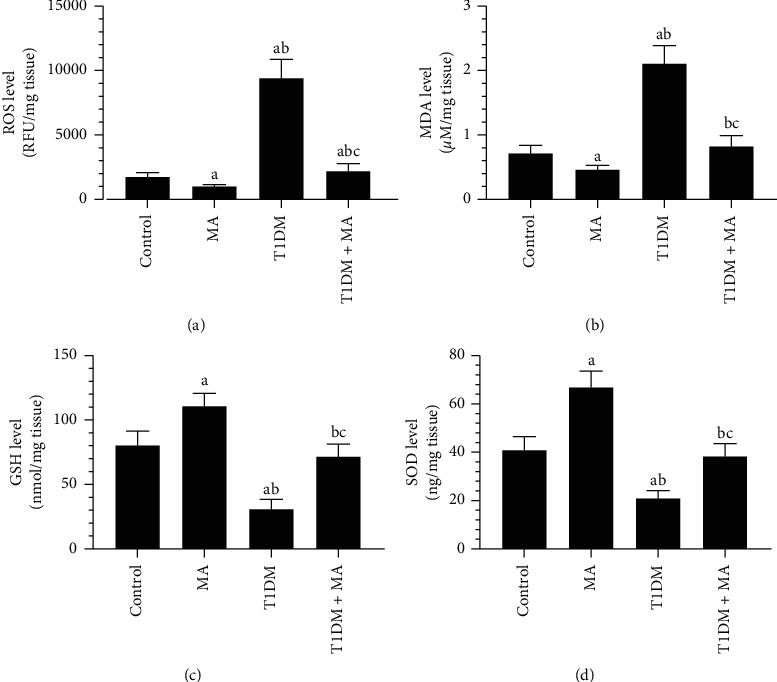
Levels of markers of oxidative stress in the retina of all groups of rats. Data were analyzed by 1-way ANOVA, and all results were presented as mean ± SD (*n* = 8). Values are considered significantly different at *P* < 0.05. ^a^: significantly different as compared to control rats. ^b^: significantly different as compared to MA-treated rats. ^c^: significantly different as compared to T1DM-induced rats.

**Figure 5:**
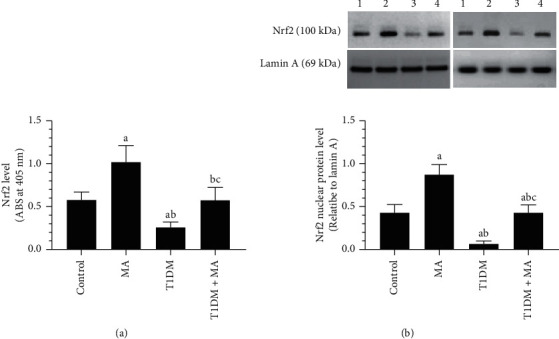
The nuclear levels of Nrf2 as detected by ELISA and by western blotting in the retina of all groups of rats. Data were analyzed by 1-way ANOVA, and all results were presented as mean ± SD (*n* = 8). Values are considered significantly different at *P* < 0.05. ^a^: significantly different as compared to control rats. ^b^: significantly different as compared to MA-treated rats. ^c^: significantly different as compared to T1DM-induced rats.

MA-treated rats had significantly lower levels of ROS and MDA that were coincided with a significant increase in the total levels of GSH and SOD, as well as the nuclear levels and protein expression of Nrf2 as compared to control rats (Figures 4(a)–4(d)and Figures 5(a) and 5(b)). On the opposite, T1DM model rats showed a significant increase in the total levels of MDA and ROS with a parallel decrease in the total levels of GSH and ROS, and in the nuclear levels and protein expression of Nrf2 as compared to control rats (Figures 4(a)–4(d) and Figures 5(a) and 5(b)). Level of ROS and MDA were significantly decreased but homogenate and protein nuclear levels of Nrf2 and total levels of GSH and SOD were significantly decreased in T1DM + MA as compared to T1DM-induced rats (Figures 4(a)–4(d) and Figures 5(a) and 5(b)).

### 3.6. MA Attenuates the Increase in the Bax/Bcl2 Ratio and the Activation of Caspase-3 in the Retinas of T1DM-Induced Rats

**Figure 6:**
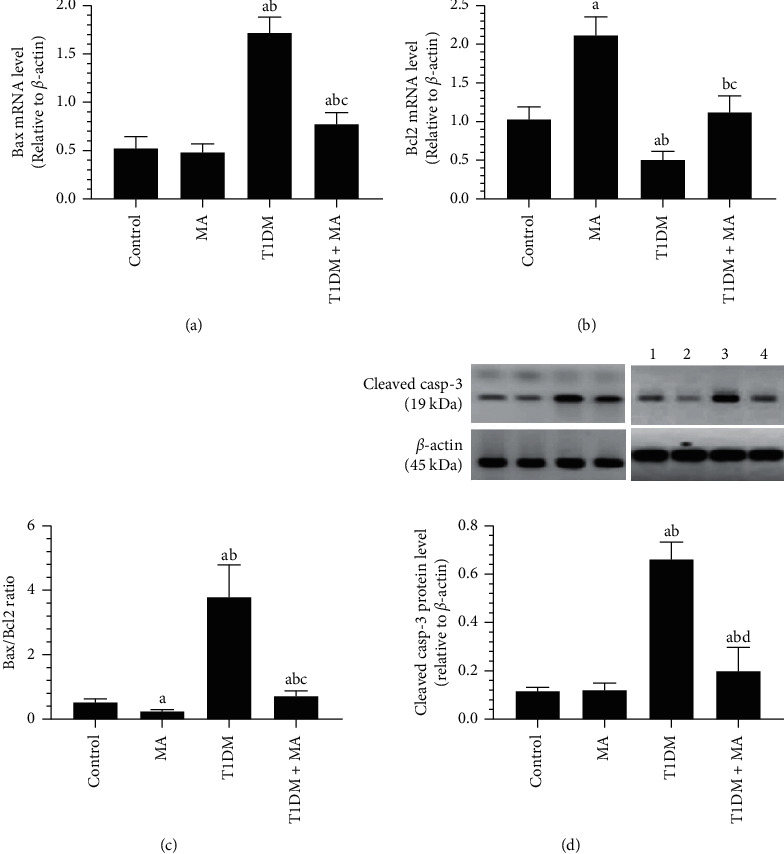
mRNA level of Bax and Bcl2 and their ratio, as well as the total protein levels of cleaved caspase-3 as detected by western blotting in the retina of all groups of rats. Data were analyzed by 1-way ANOVA and all results were presented as mean ± SD (*n* = 8). Values are considered significantly different at *P* < 0.05. ^a^: significantly different as compared to control rats. ^b^: Significantly different as compared to MA-treated rats. ^c^: Significantly different as compared to T1DM-induced rats.

mRNA levels of Bax and Bcl2, and protein levels of cleaved caspase-3 were not significantly different between the control and MA-treated rats (Figures 6(a) and 6(d)). However, mRNA levels of Bcl2 were significantly reduced leading to a significant reduction in the Bax/Bcl2 ratio in the MA-treated rats as compared to control rats (Figures 6(b) and 6(c)). In T1DM-induced rats, a significant decrease in the mRNA levels of Bcl2 with a concomitant increase in mRNA levels of Bax, Bax/Bcl2, and protein levels of cleaved caspase-3 was observed as compared to control rats, and it was reversed in the T1DM + MA-treated rats (Figures 6(a)–6(d)).

## 4. Discussion

The retina is one of the most metabolically active tissues in the human body and is one of the earliest organs affected by hyperglycaemia [9]. The sustained hyperglycaemia leads to microvascular, hemodynamic, and neural alterations that lead to DR. The mechanisms underlying DR are multifactorial but include at least oxidative stress, inflammation, angiogenesis, ischemia, and neural apoptosis [13, 18, 39]. In this study, we are providing the first evidence in the literature that MA is a neuroprotective agent that may prevent T1DM-induced DR. In addition, we are showing that the mechanism of protection involves suppressing ROS, inflammatory cytokine generation, VGEF, intrinsic cell death, upregulation of antioxidants, and inhibition of NF-*κ*B p65. However, the observation that MA upregulated levels of GSH and SOD with the concomitant nuclear activation of Nrf2 in both the control and diabetic eye suggests that the activation of Nrf2/antioxidant axis is the main mechanism of protection.

STZ is the most common drug to induce T1DM in rats and is associated with similar clinical manifestations to those seen in diabetic individuals [36]. STZ causes oxidative destruction of the pancreases, which leads to hyperinsulinemia and sustained hyperglycaemia [36]. Besides, STZ injection is associated with hepatic and renal toxicities [40, 41]. In addition, treatment with STZ is associated with typical symptoms of DM including weight loss, polyphagia, polyuria, and polydipsia. In this regard, the increase in food intake in DM is largely attributed to the associated hypoinsulinemia and hyperleptinemia [42]. However, the increased wasting the fat stores and the reliance on fatty acids as a fuel leads is the major cause of weight loss in T1DM patients [42]. All these clinical signs were also seen in our animal model 6-month post-STZ injection, thus validating T1DM in these rats.

On the other hand, chronic administration of MA to T1DM rats significantly lowered fasting glucose and partially increased fasting insulin levels. These results could explain the significant parallel improvement in body weights, despite the reduction in food intake and suggest that MA is a potent anti-diabetic molecule that could prevent T1DM complications by decreasing circulatory glucose levels and improving those of insulin. In addition, MA significantly lowered fasting serum glucose levels in the control rats without affecting the levels of insulin, confirming its hypoglycaemic effect. These data are not novel and are supported by many similar previous observations. In support, the hypoglycaemic effect of MA has been also shown in mice' and rats' animal models of both T1DM and T2DM, which were attributed to multiple mechanisms including suppressing intestinal glucose absorption (i.e., downregulating SGLT1 and GLUT2 and inhibiting *α*-glucosidase and *α*-amylase), inhibiting glycogenolysis via deactivating the glycogen phosphorylases enzymes, and improving peripheral and hepatic glucose signaling [6, 43–50]. On the other hand, although few studies were performed, the significant increase in insulin levels in our study could be explained by the antioxidant and preoperative protective effect of MA on the survival of pancreatic *β*-cells in these diabetic rats. This can be further supported by the significant increase in the structure of the islets of the Langerhans and the increase in the number of *β*-cells in the pancreases collected from the T1DM rats that received MA. In addition, the antioxidant potential of MA is well reported in several organs of diabetic and nondiabetic animal models [31, 34, 51]. However, partially unlike our data, Mkhwanazi et al., (2014) have shown that the administration of a similar dose of MA could alleviate fasting hyperglycaemia with a nonsignificant trend to increase plasma insulin levels. Such disagreement could be explained by the variations in the experimental design where those authors have started MA treatment 7 days post-STZ as compared to 3 days in our study. Also, another cause of variation could be attributed to the treatment period where they just administered MA for 5 weeks compared to 24 weeks in our study.

Oxidative stress, defined as an imbalance between the cellular antioxidant systems and ROS [52], has been largely known to be the major mechanisms underlying all damaging effects and pathogenies of DR [20–24]. In the retina, oxidative stress causes basement membrane thickness and induces BRB damage, hypoxia, inflammation, apoptosis, angiogenesis, and neovascularization [13, 18]. However, antioxidant therapy is sufficient to prevent retinal damage by attenuating all these mechanisms [19–24, 39]. In most cells, the antioxidant system is composed of the nonenzymatic components (i.e., GSH) and antioxidant enzymes including SOD, catalase, glutathione peroxidase (GPx), glutathione reductase (GRx), and others [53]. Excessive production of ROS not only induces lipid peroxidation by the excessive production of MDA, but also leads to cell damage by overwhelming antioxidants and promoting mitochondria damage, DNA breaks, inflammation, and apoptosis [53]. On the other hand, Nrf2 is described as the major antioxidant transcription factor that stimulates the synthesis and recycling of GSH and the upregulation of phase II antioxidant enzymes including heme-oxygenase-1 (HO-1), quinone dehydrogenase 1 (NQO1), SOD, and catalase [54]. Under normal conditions, Nrf2 is trapped in the cytoplasm and undergoes cytoplasmic proteasome ubiquitination by binding to keap-1 [54]. However, under stress, ROS causes the phosphorylation, redox modification, and dissociation of keap-1, thus allowing the nuclear translocation of Nrf2 to initiate the transcription process [55].

Oxidative stress has been identified as the major mechanism underlying DR and is increased production of ROS, depletion of antioxidants, and suppression of Nrf2 [10, 56, 57]. Within this view, hyperglycaemia can induce massive quantities of ROS in the retina by promoting glucose auto-oxidation, impairing mitochondria oxidative phosphorylation, and activating numerous ROS generator mechanisms such as NADPH oxidase, protein kinase c (PKC), hexosamine, and AGE/RAGEs pathways [13]. Also, hyperglycaemia can stimulate the production of ROS by decreasing the retinal levels of GSH through activating the polyol pathway [13]. In addition, hyperglycaemia and ROS can amplify ROS by suppressing the expression of the transactivation of Nrf2 via acetylation and methylation [13]. Supporting these studies, diabetic rats of this study showed higher levels of ROS, increased production of MDA, reduced levels of GSH and SOD, and decreased nuclear accumulation and levels of Nrf2 in their retinas. These changes were prevented by the administration of MA. Although these effects could be attributed to the previously discussed hypoglycaemic potential of MA, treatment with MA also stimulated Nrf2 and levels of GSH and SOD and reduced the generation of ROS and MDA in the retina of control rats too, thus owing a potent independent antioxidant effect.

Indeed, numerous line of evidence has confirmed the antioxidant power of MA. In this regard, review studies have shown that the antioxidant potential of MA against multiple organ damage is mediated by suppressing ROS generation, lipid peroxidation, upregulation of antioxidants, and activation of Nrf2 [31]. Besides, MA has been also shown that MA has potent peroxyl-radical scavenging and chelating abilities and was able to prevent the generation of ROS by acting similarly to catalase to reduce the generation of hydrogen peroxides (H2O2) [31]. In particular, MA prevented DM-induced renal, hepatic, and cardiac damage by reducing ROS and lipid peroxidation through the stimulating cellular levels of SOD and glutathione peroxidase (GPX) [34]. In addition, MA prevented ROS generation and stimulated antioxidant expression in CCL4-stimulated macrophages [26]. In the same line, MA protects against oxidative stress-induced vascular smooth muscle (VSM) degeneration by scavenging ROS, upregulating antioxidants, and activating the Nrf2/HO-1 pathway [28]. It also reduced the oxidation of low-density lipoprotein (LDL-c) [58]. Furthermore, MA inhibited the generation of ROS in the endothelial cells and pericytes after an ischemia/reperfusion (I/R) episode [32].

Nonetheless, DR is also associated with severe inflammation that it could exaggerate the oxidative stress response and induce tissue apoptosis [10, 13, 52]. Oxidative damage and inflammation are closely related to each other where a positive cross-talk exists between the two [59, 60]. NF-*κ*B is the major inflammatory transcription factor that stimulates cell inflammation and ROS generation by upregulating inflammatory cytokines and mediators such as TNF-*α*, IL-6, cyclooxygenase-2 (COX-2), intracellular nitric oxide synthase (iNOS), and IL-1*β* and is mainly activated through the Toll-like receptor-type 4 (TLR4) [61]. Other cellular factors that can also stimulate NF-*κ*B in the diabetic retina include growth factors, AGEs, and oxidized lipids and proteins, [13, 18]. However, the inflammatory picture in the diabetic retina is characterized by increased immune cell infiltration, leucocytes, microglial and complement system activation, higher levels of adhesive molecules levels (ICAM-1 and P-selectin) and inflammatory cytokines (COX-2, TNF-*α*, IL-1), and hyper-activated NF-*κ*B [10, 14, 62].

In this study, and associated with the oxidative stress response, retinas of diabetic rats also showed a significant increase in the nuclear activities of NF-*κ*B and the levels of ICMA, TNF-*α*, and IL-6, thus suggesting that inflammation is an indispensable mechanism during the progression of DR. On the other hand, MA significantly reversed this in the retina of diabetic rats but did not affect the levels/activities of all these markers in the retinas of control rats. Based on these data, we have concluded that the anti-inflammatory effect of MA is more likely secondary to its antioxidant potential and suppresses the generation of the upstream trigger, ROS. Supporting our data, the anti-inflammatory effect of MA has been largely described in various disorders and was shown to parallel with its antioxidant effects [31]. In this context, MA protected against DM-induced cardiomyopathy by suppressing NADPH oxidase and NF-*κ*B [31]. It also prevented alcohol-induced hepatic damage and osteoclast genesis by suppressing NF-*κ*B and immune cell infiltration [63, 64]. In the same line, MA also prevented ischemia/reperfusion (I/R)-induced inflammation and oxidative damage in the endothelial cells and pericytes by reducing ROS generation and the expression of adhesive molecules (i.e., E-selectin, ICAM, and VCAM), and inhibiting NF-*κ*B [32]. Also, MA prevented lipopolysaccharide (LPS)-induced inflammation in the macrophages, as well as in the cortical astrocytes by reducing the generation of ROS, H2O2, and NO, inhibiting iNOS and COX2, and suppressing NF-*κ*B and the production of TNF-*α* and Il-6 [30, 65]. Furthermore, the anti-inflammatory effect of MA in Raji cell lymphoma was mediated by suppressing NF-*κ*B, COX2, activator protein (AP-1), and NF-*κ*B [66].

Oxidative stress and inflammatory cytokines can activate cell apoptosis leading to organ damage [67]. In the cells, the caspase family are the most known enzymes that initiate cell death [68]. Caspases-8 and caspase-9 are the best known initiator caspase that stimulates the cleavage and the activation of the executioner caspases (i.e., caspases-3 and caspase-9) [68, 69]. In general, cell apoptosis is involving both external and intrinsic (mitochondria-mediated) cell death [68, 70, 71]. The activation of the death receptors by the inflammatory TNF-*α* stimulates extrinsic and intrinsic cell death by the upregulating caspases-8 [68, 71]. On the other hand, intracellular signaling such as ROS can initiate the intrinsic cell apoptosis by upregulating p53/Bax, downregulating Bcl2, and stimulating the Bax, which eventually leads to mitochondria damage and the release of cytochrome-c [68].

Intrinsic cell death is the most common modality of cell apoptosis in the diabetic eye and is characterized by increased expression of Bax and caspase-3, cleavage of caspase-3, and downregulation of Bcl2 [37, 72]. This has been also confirmed in the retinas of diabetic rats of this study too. However, the ability of MA to inhibit the transcription of Bax and protein levels of cleaved caspase-3, as well as to upregulate levels of Bcl2, was our strongest available evidence for the anti-apoptotic effect of MA. This could be explained by the previously discussed antioxidant and anti-inflammatory effects of MA. Interestingly, we also found a stimulatory effect of MA on the transcripts of Bcl2 in the retinas of control rats too, which could be explained by the concomitant activation of Nrf2. Indeed, and independent of its antioxidant effect, MA is a potent anti-apoptotic factor due to its ability to increase the cellular availability of Bcl2 [73]. In the same line with our findings, the antioxidant and anti-inflammatory effects of MA were referred to as its anti-apoptotic effect through inhibiting both extrinsic and intrinsic apoptosis in a variety of tissues and cells, *in vivo* and *in vitro* [13, 26, 28, 31, 34, 51, 55, 56].

Overall, these data suggest that MA could alleviate DR in T1DM rats by suppressing oxidative stress, inflammation, and apoptosis. Despite these findings, this study still has some limitations. Of importance, our data are still observational, and further evidence at the levels of transgenic animals or inhibiting or knocking down Nrf2 is required to confirm these data. This is highly recommended. Besides, MA protected against diabetic nephropathy and ischemia/reperfusion injury by activating the SIRT1/AMPK axis [74, 75]. Since hyperglycaemia inhibits Nrf2 by deacetylation through suppressing SIRT1, it could be possible that MA stimulates Nrf2 by activating SIRT1 directly or through its hypoglycaemic effect. Digging down this mechanism in future studies will open the window for us to reveal the precise mechanism of action.

In conclusion, our data remain very interesting and clearly show a protective effect of MA against STZ-induced DR and reveal that the mechanism of protection involves hypoglycaemic and antioxidant potential. Further preclinical and translational studies are encouraged to confirm these effects, which could provide a golden novel therapy in patients.

## Figures and Tables

**Table 1:** Changes in final body weights, food and water intake, and serum levels of fasting glucose and insulin levels in all groups of rats.

Parameter	Control	MA	T1DM	T1DM + MA
Final body weight (g/rat)	454 ± 34.4	438.6 ± 46.3	343.3 ± 22.6^**ab**^	421.3 ± 32.5^**c**^
Average weekly food intake (g/group)	1783 ± 231	1694 ± 189	2753 ± 331^**ab**^	1892 ± 254^**c**^
Weekly water intake (ml/group)	2246 ± 398	2381 ± 254	3779 ± 378^**ab**^	2667 ± 392^**c**^
Serum glucose (mg/dl)	107.4 ± 7.8	93.4 ± 5.6^**a**^	341.5 ± 17.6^**ab**^	136.3 ± 10.2^**abc**^
Serum insulin (*µ*IU/ml)	4.87 ± 1.04	5.1 ± 1.22	1.94 ± 2.9^**ab**^	2.67^**abc**^

Data were analyzed by 1-way ANOVA, and all results were presented as mean ± SD (*n* = 8). Values are considered significantly different at *P* < 0.05. ^**a**^: significantly different as compared to control rats. ^**b**^: significantly different as compared to MA-treated rats. ^**c**^: significantly different as compared to T1DM-induced rats.

**Table 2:** Morphometric quantitative analysis of the thickness of different layers in the retina of all groups of rats.

Parameter	Control	MA	T1DM	T1DM + MA
Distance between ILM and OLM (*µ*m)	265 ± 15.8	258 ± 22	160 ± 16.5^**ab**^	248 ± 16.9^**c**^
OLN thickness (*µ*m)	72.3 ± 7.4	77.2 ± 9.7	45.6 ± 5.4^**ab**^	74.3 ± 8.1^**c**^
INL thickness (*µ*m)	51.2 ± 4.5	49.6 ± 4.7	25.6 ± 3.4^**ab**^	44.3 ± 6.5^**c**^
No. of cells in the GCL /section	28.4 ± 3.2	27.6 ± 4.3	10.4 ± 2.1^**ab**^	22.3 ± 3.3^**abc**^
No. of cells in the INL	278.3 ± 37.6	294 ± 33.5	129 ± 20.5^**ab**^	238.4 ± 29.8^**abc**^
No. of cells in the ONL	450.2 ± 52.3	439 ± 41	345 ± 31.3^**ab**^	462 ± 45.3^**c**^

Data were analyzed by 1-way ANOVA, and all results were presented as mean ± SD (*n* = 8). Values are considered significantly different at *P* < 0.05. ^**a**^: significantly different as compared to control rats. ^**b**^: significantly different as compared to MA-treated rats. ^**c**^: significantly different as compared to T1DM-induced rats.

## Data Availability

The data that support the findings of this study are available from the corresponding author upon request.
